# Gliadin Peptides as Triggers of the Proliferative and Stress/Innate Immune Response of the Celiac Small Intestinal Mucosa

**DOI:** 10.3390/ijms151120518

**Published:** 2014-11-07

**Authors:** Maria Vittoria Barone, Riccardo Troncone, Salvatore Auricchio

**Affiliations:** 1 Department of Translational Medical Science (Section of Pediatrics), University of Naples Federico II, Via S. Pansini 5, Naples 80131, Italy; E-Mail: troncone@unina.it; 2 European Laboratory for the Investigation of Food Induced Diseases (ELFID), University of Naples Federico II, Via S. Pansini 5, Naples 80131, Italy; E-Mail: salauric@unina.it

**Keywords:** celiac disease, gliadin, gliadin peptides, peptide 31–43 (P31–43), innate immunity, epithelial growth factor/epithelial growth factor receptor (EGF/EGFR), interleukin-15/interleukin-15 receptor-α (IL-15/IL-15R-α), cellular stress, actin, proliferation

## Abstract

Celiac disease (CD) is a frequent inflammatory intestinal disease, with a genetic background, caused by gliadin-containing food. Undigested gliadin peptides induce innate and adaptive T cell-mediated immune responses. The major mediator of the stress and innate immune response to gliadin peptides (*i.e.*, peptide 31–43, P31–43) is the cytokine interleukin-15 (IL-15). The role of epithelial growth factor (EGF) as a mediator of enterocyte proliferation and the innate immune response has been described. In this paper, we review the most recent literature on the mechanisms responsible for triggering the up-regulation of these mediators in CD by gliadin peptides. We will discuss the role of P31–43 in enterocyte proliferation, structural changes and the innate immune response in CD mucosa in cooperation with EGF and IL-15, and the mechanism of up-regulation of these mediators related to vesicular trafficking. We will also review the literature that focuses on constitutive alterations of the structure, signalling/proliferation and stress/innate immunity pathways of CD cells. Finally, we will discuss how these pathways can be triggered by gliadin peptide P31–43 in controls, mimicking the celiac cellular phenotype.

## 1. Introduction

Ingested food can cause tissue inflammation through different mechanisms. In the intestine, and particularly in the enterocyte, nutrients are modulators of various cellular functions and may be involved in tissue immune response and inflammation [1]. An example of an intestinal inflammatory and remodeling response of the intestine to food is the small intestinal celiac lesion induced by gluten, an alimentary protein present in wheat and other cereals. Celiac disease (CD) is characterized by inflammatory and structural changes resulting in remodeling of the small intestinal mucosa [2,3,4].

Gliadin, the major protein component of wheat and other cereals, is a peculiar protein very rich in glutamine and proline. Several gliadin peptides are recognized by T-cells (TC) of the celiac intestine, and can induce the adaptive immune response, but most of them are digested by gastric, pancreatic and intestinal proteases. Only two main peptides remain undigested [5,6,7,8]: the 33-mer (P55–87) and the 25-mer (P31–55). Consequently, these two peptides are the main peptides that are active in vivo in the celiac intestine after gluten ingestion.

The inflammation of the intestinal mucosa is due not only to the adaptive but also to the innate immune responses to wheat gliadin. The A-gliadin 33-mer that is deamidated by tissue transglutaminase (tTG), binds to human leukocyte antigen (HLA) DQ2 and/or DQ8 and induces an adaptive Th1 pro-inflammatory response [9]. The P31–43 peptide, which is contained in the 25-mer, is not recognized by TC in the celiac intestine and is able to damage the celiac intestinal mucosa *in vitro* and *in vivo* [10,11,12]. Moreover, the P31–43 gliadin peptide is able to initiate both a stress [13,14] and an innate immune response [15,16] with interleukin-15 (IL-15) as a major mediator.

**Figure 1:**
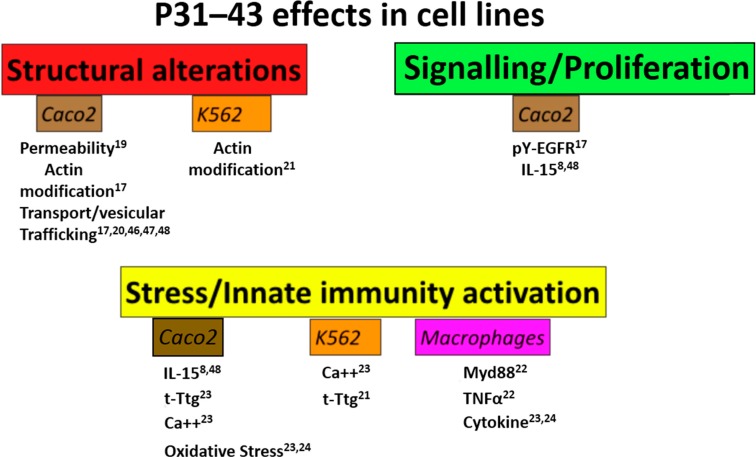
Peptide 31–43 (P31–43) has several effects on cell lines. Schematic representation of the effects of gliadin peptide P31–43 on several cell lines (Caco2, K562, Macrophages). The main effects were grouped in three sets: structural alterations (permeability, actin modifications and alterations in vesicular trafficking), signaling/proliferation (epithelial growth factor/epithelial growth factor receptor- interleukin-15/interleukin-15 receptor-α (EGF/EGFR-IL-15/IL-15R-α) activation, pY-extracellular signal-regulated kinase (pY-ERK)) and stress/innate immunity activation. In all cases there was a quantitative increase in the markers cited, although in the case of actin, the alterations were qualitative. Numbers indicate the bibliographic references.

Although the structural changes of the celiac mucosa are considered a consequence of sustained mucosal inflammation due to the Th1-TC response, recent data have shown that gliadin peptides, in particular P31–43, induce proliferation of celiac enterocytes. This process is epithelial growth factor (EGF) and IL-15 dependent, and has profound upstream effects in inducing the crypt hyperplasia, which is characteristic of the remodeling of the celiac mucosa [17,18,19]. Moreover, gliadin peptides induce alterations of structure (cell shape, actin modifications, increased permeability [19] and vesicular trafficking alterations [17,20]), signaling [17,18] and proliferation [17] and stress/innate immunity activation in several cell lines [21,22,23,24,25,26] (Figure 1).

**Figure 2:**
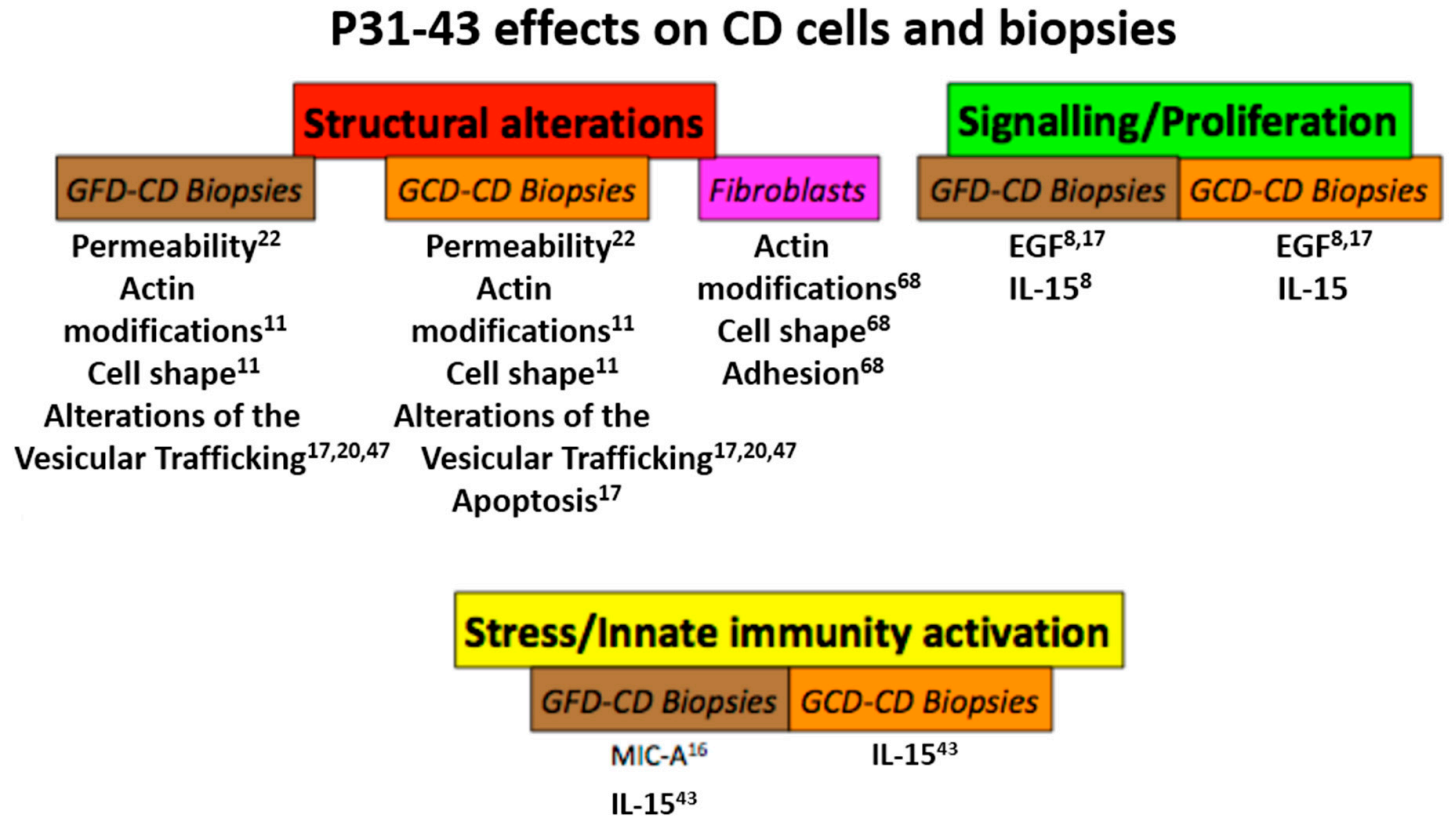
P31–43 affects cells and biopsies from celiac disease (CD) patients. Schematic representation of the effects of gliadin peptide P31–43 on CD cells and biopsies. The main effects were grouped in three sets: structural alterations (permeability, actin modifications, cell shape and transport/trafficking), signaling/proliferation (EGF/EGFR-IL-15/IL-15R-α activation, pY-ERK) and stress/innate immunity activation as shown. In all cases, there was a quantitative increase in the markers cited, although in the case of actin, the alterations were qualitative. Numbers indicate the bibliographic references. GCD: gluten-containing diet. GFD: gluten-free diet.

Taken together, these data suggest that gliadin peptides (*i.e.*, P31–43) can have several different non-T cell mediated effects, both in cell lines and cells and in biopsies from CD patients, that can be grouped into three sets: cell structural changes, including apoptosis, signaling/proliferative effects and stress/innate immunity activation (Figure 2). How these mechanisms of disease are related to the genetics of CD is unclear.

## 2. Results and Discussion

Many questions are unanswered regarding gliadin peptides, and in particular P31–43, effects on stress/innate immune response in CD. How gliadin peptides activate innate immune response? How innate immune response is related to gliadin biological effects on cells and tissues? What is the mechanism generating the stress/innate immune response? What makes the celiac cells susceptible to gliadin peptides effects? Are non-celiacs not susceptible to these effects?

In this review, we will try to answer to these questions discussing some recent data from the literature on the effects of gliadin peptides, in particular P31–43, on control and celiac cells (fibroblasts and dendritic cells) and intestinal biopsies, highlighting their relationship with IL-15 and EGF/EGFR deregulation in CD.

### 2.1. The Role of IL-15 in the Pathogenesis of CD

IL-15 exerts pleiotropic activity that ultimately results in immunoregulatory cross-talk between cells of the innate and adaptive branches of the immune response. Moreover, IL-15 can induce the proliferation of intestinal epithelial cells [27,28].

#### 2.1.1. IL-15 Expression in CD

The increase of IL-15 and IL-15R-α is a major event in the initial phases of CD [29,30,31]. IL-15 is increased in the serum [32] and in the intestinal mucosa at the level of the enterocytes and of the lamina propria [33] of celiac subjects in the active and remission phases of the disease [14,16,34].

#### 2.1.2. Role of IL-15 in the Break of Oral Tolerance to Gluten

IL-15 inhibits regulatory T-cells (TREG) [14,35] and the immunoregolatory transforming growth factor (TGF) signalling, moreover it also interferes with suppressive activity of CD4+ CD25+ FOXP3+ regulatory T cells expanded in celiac intestinal mucosa [35,36,37]. These data provide a rational for the loss of intestinal tolerance to gluten. In a transgenic mouse that expresses the human HLA class II molecule DQ8 and IL-15 in the lamina propria and not in the epithelium, gliadin and retinoic acid treatment can reproduce an early stage of CD with interferon (INF) γ producing anti-gliadin TC, anti-gliadin and anti-tissue transglutaminase antibodies and intraepithelial lymphocytosis in the absence of villous atrophy [38]. In conclusion IL-15 in the lamina propria can be responsible for the loss of oral tolerance to gluten in CD.

#### 2.1.3. Role of IL-15 in the Damage of the Enterocyte in the CD Intestine

IL-15 stimulates the effector functions of intestinal epithelial lymphocytes (IEL) in active celiac disease with the production of γ-INF and perforin-granzyme-dependent cytotoxicity against the enterocytes [16,33].

In fact, the IL-15 in the intestinal mucosa of celiac disease up-regulates the expression in IEL of two receptors (CD94 and NKG2D) and in the enterocytes of their ligands (MICA and HLAE), resulting in apoptosis [16,29,39,40].

Transgenic mice that express human IL-15 specifically in the enterocytes develop villous atrophy, crypt proliferation and severe duodenal-jejunal inflammation with massive accumulation of NKG2D-expressing CD8+ intraepithelial lymphocytes in the affected mucosa, reproducing the intraepithelial inflammation and damage typical of CD in a gluten-independent way. The blockage of IL-15 signaling with antibodies leads to a reversal of the intestinal damage [41]. These mice also display autoantibodies, including antibodies against tTG2, and extensive lamina propria plasmacytosis, features that are characteristic of CD, suggesting that epithelial expression of IL-15 drives both the CD8+ TC and B cell pathologic effects seen in CD [42].

IL-15 is therefore a major mediator of the stress and innate immune response to gliadin peptides (e.g., P31–43) and a central regulator of celiac disease immunopathology. For a recent review on this subject see the publication of Abadie and Jabrì [43].

Although in the past it was suggested that gliadin peptides could promote IL-15 expression by IEC (Intestinal Epithelial Cells) [15], the mechanisms responsible for triggering IL-15 up-regulation in CD have not been well defined [43]. More recently some reports, which we will review in this paper, have begun to unveil a possible mechanism of IL-15 activation by gliadin peptides in enterocytes.

### 2.2. Role of P31–43 in Enterocyte Proliferation, Structural Changes and Innate Immune Response in CD Mucosa: EGF and IL-15

Damage to the intestinal mucosa in CD is mediated both by inflammation due to the adaptive and innate immune response to gliadin and by proliferation of crypt enterocytes as an early alteration of CD mucosa causing crypt hyperplasia [2,3,4]. The celiac intestine is characterized by an inversion of the differentiation/proliferation program of the tissue, with a reduction in the differentiated compartment, up to complete villous atrophy, and an increase in the proliferative compartment with crypt hyperplasia [18,44,45].

Gliadin peptides and P31–43 induce cell proliferation and actin rearrangements [8,17] in various cell lines, mimicking the effect of EGF [17]. Gliadin peptides enhance the EGF pathway by increasing EGFR and ERK phosphorylation with consequent actin remodeling and proliferation. The activation of the EGFR pathway is a consequence of delayed endocytosis and delayed inactivation of the EGF receptor (see mechanisms paragraph) [17,46,47].

P31–43 is also able to enhance proliferation of the celiac enterocytes in an EGF-dependent way and to delay the trafficking and degradation of EGFR at the epithelial level, suggesting a role for EGFR activation in CD, particularly in determining crypt hyperplasia and the tissue remodeling of the celiac intestine. Persistent epithelial cell proliferation leads to inhibited maturation and differentiation of epithelial cells and loss of the normal villous structure [17]. Gliadin peptide- and P31–43-induced proliferation of CD crypt enterocytes (and Caco2 cells) is dependent not only on EGFR but also on IL-15 activity, as demonstrated by the inhibitory effect of antibodies neutralizing EGFR and IL-15 and by silencing experiments [8,48].

In particular, in Caco2 cells, proliferation can be induced by both IL-15 and EGF and is dependent on interplay between EGFR and IL-15R-α. The cooperation is mediated by a complex between IL-15R-α and EGFR which is increased and activated by each ligand. The signaling, beginning with the complex of the two receptors can be activated by EGF and IL-15, with each of them able to stimulate its own and the other receptor. Moreover, both EGF and IL-15 can induce transcriptional activation of each other. P31–43, which induces enterocyte proliferation and the activation of IL-15 in CD, increases the complex, the activation and the downstream signaling of both receptors together with the transcripts of both ligands. These data show that the proliferation of enterocytes can be regulated by interplay between growth factors (EGF) and cytokines (IL-15) and that P31–43 can stimulate growth and the innate immune response by employing such cooperation [8].

### 2.3. Mechanisms of IL-15 and EGF Up-Regulation in CD Induced by P31–43: Role of Vesicular Trafficking

The mechanisms through which P31–43 might induce the celiac intestine innate immune response and EGF- and IL-15-mediated enterocyte proliferation have recently been investigated. Recent evidence [17,47,48] points to an effect of P31–43 on the endocytic compartment.

**Figure 3:**
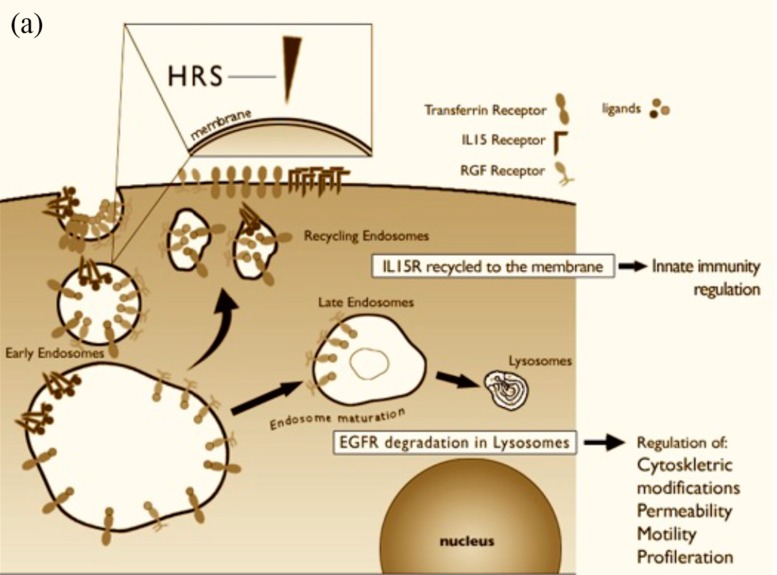

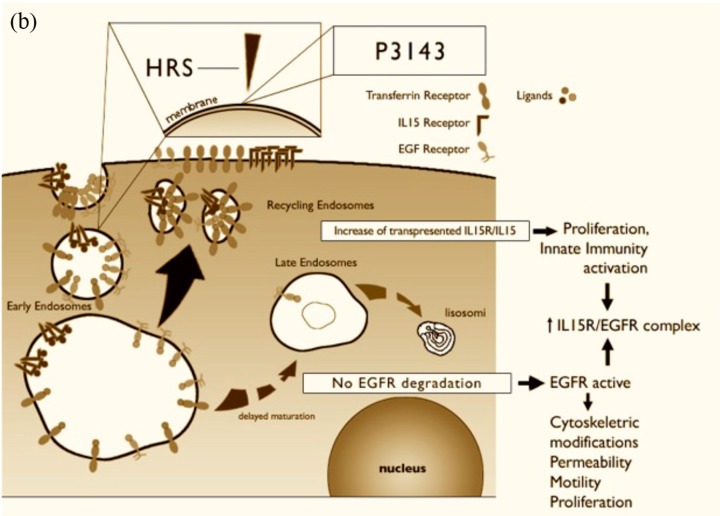
(**a**) Schematic representation of receptor-mediated endocytosis. Trafficking of IL-15R-α, EGFR and transferrin in normal cells. The ligands bind to their receptors on the cell surface. The ligand/receptor complexes are internalized by a process of endocytosis and enter into vesicles “early”. The protein hepatocyte growth factor regulated substrate (HRS) regulates the maturation of endosomes from early to late. If HRS is not properly localized on the membrane of early endosomes, vesicular trafficking is altered. IL-15 and transferrin entering the compartment vesicular recycling are transported to the cell membrane. Receptor tyrosine kinases, such as EGFR, are only partially recycled because their principal destiny is to be transported in the late vesicular compartments to be degraded in lysosomes. When these receptors are in the early compartment, they can still signal within the cell. These transport phenomena within the cell regulate the duration of the activation of the receptors regulating processes essential for cell function, such as activation of innate immunity, cell proliferation, regulation of the actin cytoskeleton, motility and permeability. Alterations in the maturation of endosomes may therefore impair the functionality of the cell in many different ways; and (**b**) Effect of P31–43 on vesicular trafficking of IL-15R-α, EGFR and transferrin within the cells. The peptide P31–43, due to a sequence homology with HRS, interferes with its correct localization at the level of early endosomes, with a consequent slowing of the maturation of endosomes from early to late, prolonged activation of EGFR, and an increase of transferrin on the cellular membrane and trans-presented IL-15. The main biological consequences at the level of enterocytes of the altered trafficking of these receptors are: Increased proliferation, an alteration in permeability, alterations in the cytoskeleton and cell shape, and an increase in the innate immune response.

Endocytosis has many effects on signaling; in fact, signaling pathways and endocytic pathways are regulated in a reciprocal manner. It is now widely accepted that the “endocytic matrix” is a master organizer of signaling, governing the resolution of signaling in space and time. Consequently, endocytosis affects several cell functions, ranging from proliferation to actin organization, cell motility and stress/innate immunity activation [49,50] (Figure 3a,b).

P31–43 is strikingly similar to a region of hepatocyte growth factor regulated substrate (HRS) kinase, a key molecule regulating endocytic maturation, which is localized on the membranes of early endocytic vesicles [51] (Figure 3b).

The sequence similarity between gliadin peptide P31–43 and HRS is in a small area of the proline/glutamine rich domain of HRS. The COOH- terminal of HRS contains a clathrin-binding domain that binds clathrin to clathrin-coated vesicles [52] and is one of the domains needed to localize HRS to the vesicle membranes [53,54,55,56]. Both in Caco2 cells and in the celiac enterocytes, P31–43 localizes in the early endosome and delays vesicular trafficking.

In Caco2 cells, P31–43 interferes with the correct localization of HRS at the level of the early endosomes, interfering with HRS-mediated maturation of early endosomes. By interfering with the localization to the endocytic membrane of HRS, P31–43 induces two important effects: (a) it delays endocytic maturation and (b) it alters the recycling pathway. By delaying the maturation of endocytic vesicles, P31–43 reduces the degradation of EGFR and other receptor tyrosine kinases (RTKs) that are endocytosed in these vesicles, and prolongs their activation, resulting in increased proliferation, actin remodeling and other biological effects. The alteration of the recycling pathway is able to direct more transferrin receptor [48] and IL-15R-α to the cell surface, allowing more IL-15/IL-15R-α trans-presentation in epithelial cells. Moreover, the trans-presented IL-15 is able to activate IEL *in vitro* in an IL-15-dependent way, demonstrating that the effects of P31–43 on enterocytes can activate signaling in lymphocytes. Interestingly, more IL-15R-α is expressed in CD enterocytes and in patients on a gluten-free diet [31], indicating that in the CD mucosa, a constitutive alteration of IL-15R-α trafficking could be present (see below).

The production of IL-15 is tightly controlled at multiple levels, not only at the level of intracellular trafficking but also of transcription and translation [43]. P31–43 increased IL-15 mRNA levels only after prolonged incubation, whereas the increase of the trans-presented IL-15/IL-15R-α complex on the cell surface was an early effect [48].

By increasing the synthesis of IL-15 and the amount of the cytokine that is trans-presented to the neighboring cells, P31–43 affects both enterocyte proliferation, which is EGFR-IL-15 dependent, and the activation of innate immunity [48].

We would like now to review the hypothesis that the effects of gliadin and P31–43 on endocytosis, a pathway nodal to many cell functions, could explain the sensitivity to gluten of CD cells.

### 2.4. Constitutive Alterations in CD Cells

**Figure 4:**
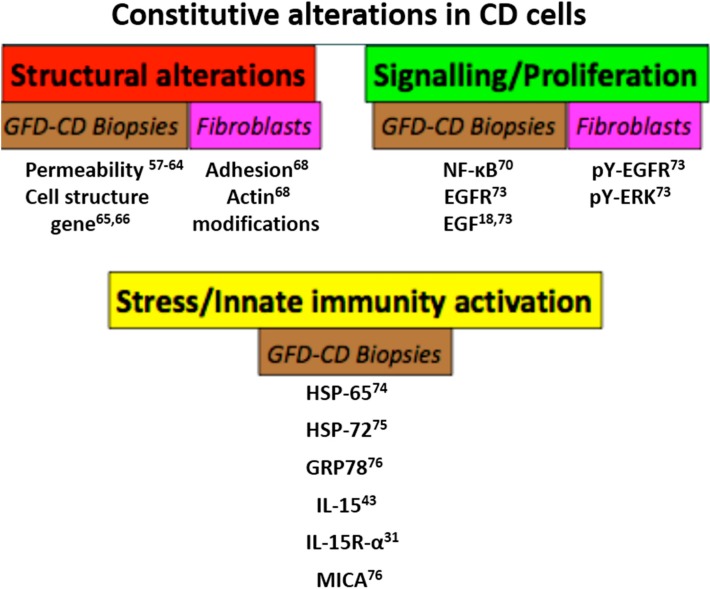
Constitutive alterations in cells and biopsies from CD patients. Schematic representation of constitutive alterations of CD cells and biopsies. These alterations have been described in gluten-free diet (GFD) CD patient cells and biopsies. The main constitutive alterations found in GFD CD cells and biopsies were grouped in three sets: Structural alterations (permeability, actin modifications, adhesion), signaling/proliferation (EGF/EGFR activation, NF-κB, pY-ERK) and stress/innate immunity activation as shown. In all cases there was a quantitative increase in the markers cited, although in the case of actin, the alterations were qualitative. Numbers indicate the bibliographic references.

Recent observations suggest an effect of P31–43 on the maturation and function of early endocytic vesicles and consequently on EGFR signaling, enterocyte proliferation and IL-15 trans-presentation and synthesis [17,46,47,48,51]. However, the explanation for why the stress/innate immune and proliferative responses to certain gliadin peptides (e.g., P31–43) in the CD intestine are so intense and disruptive has not been elucidated. For this reason, it is interesting to review the recent literature regarding constitutive alterations in CD biopsies and cells. Several reports note constitutive, gluten-independent alterations of the CD cells. They have been studied in the normalized intestinal biopsies of patients in the remission phase of the disease on a gluten-free diet and in cells obtained from tissues far away from the intestine, the primary site of inflammation (Figure 4).

#### 2.4.1. Structural Alterations

Different studies have identified the importance of the disruption of the integrity of the epithelial layer in CD. One of the first structural alterations identified is the alteration of the cell-to-cell junctional complexes that regulate intestinal permeability. Patients show enhanced intestinal permeability and altered tight junction (TJ) morphology, and these disruptions persist in patients who are on a gluten-free diet (GFD) with a normalized intestine, suggesting that permeability may play a driving role in the development of CD [57,58,59,60,61]. Moreover, epithelium integrity is impaired in the early stage of the disease [62]. Polymorphisms in the TJ genes *PARD3* and *MAGI2* have been associated with disease susceptibility in a Dutch cohort [63]. Interestingly, PPP2R3A, implicated in the negative control of cell growth, division and TJ regulation, remains down-regulated at the intestinal level in patients on a gluten-free diet [64]. These observations suggest a role for this pathway in the pathogenesis of CD.

Recent genetic studies point to the importance of polymorphisms of CD genes that are involved in actin remodeling and cell adhesion. Among these, the *LPP* gene presents the strongest non-HLA association signal, mapped in intron 2 [65]. More recently, it has been suggested that deregulation of transcription binding properties, due to single point mutations, might be the causal mechanism underlying the association of CD with the LPP region [66]. The LPP protein localizes to focal adhesions, which are the site of membrane attachment to the extracellular matrix and cell-cell contact [67]. A constitutive alteration of LPP sub-cellular distribution together with alterations of cell shape, actin cytoskeleton and focal adhesion has been demonstrated in CD fibroblasts from GFD patients [68]. Moreover, cell shape and actin rearrangements are altered in CD dendritic cells from GFD patients.

Taken together, these data indicate that structural alterations are present in CD cells independent of gluten.

#### 2.4.2. Signaling and Proliferation

Alterations in signaling pathways and cell proliferation have been demonstrated in CD biopsies and cells (enterocytes, skin fibroblasts, dendritic/monocytes) of patients on a GFD.

##### NF-κB Pathway

The NF-κB pathway is constitutively altered in CD, with more than 20 components of the pathway increased in GFD-CD biopsies. Most of the mRNA over expressed in GFD-CD was central to the regulation of the pathway [69]. Interestingly, two key mediators of the NF-κB pathway, TNFAIP3, have CD-associated gene variants [70]. It is widely accepted that NF-κB is a key regulator of inducible gene expression in the immune system. Both innate and adaptive immune responses, as well as the development and maintenance of the cells and organs that comprise the immune system are, at multiple stages, under the control of the NF-κB family of transcription factors. Moreover, NF-κB is responsible for the transcription of genes encoding a number of pro-inflammatory cytokines and chemokines [71]. It has also been shown that NF-κB is a major mediator of IL-15 [72], which, among its many pleiotropic effects, is also able to decrease claudin-2 levels in epithelial tight junction structures and leads to augmented paracellular permeability, a phenomenon that is relevant and persistent in CD.

##### EGF Receptor/Ligand System

Constitutive activation of the EGF receptor/ligand system is also present in CD enterocytes [18]. Increases in EGFR protein levels, EGF mRNA, the downstream effector molecule ERK and proliferation, which is ERK-dependent, have been found in enterocytes from normal biopsies of GFD-CD patients [73].

#### 2.4.3. Stress/Innate Immunity Activation

##### Cellular Stress

Cellular stress has been implicated in the early events of the disease, in particular in the epithelium [13,14]. Heat Shock Protein-65 (HSP-65) is increased in CD enterocytes before they develop the disease, indicating that epithelial stress may play a role in the pathogenesis of CD [74]. An alteration of this pathway was confirmed later with the observation that HSP-72 increased [75]. More recently, a marker of endoplasmic reticulum (ER) cellular stress, the molecular chaperone glucose regulate protein 78 (GRP78), the master negative regulator of the unfolded protein response (UPR), was found increased in enterocytes with moderate and severe enteropathy and after at least two years on a gluten-free diet. Remarkably, the increased peri-nuclear GRP78 aggregates co-localize with increased MICA/B+ in CD enterocytes, linking cellular stress and innate immunity in CD [76].

##### IL-15/IL-15R-α

IL-15 is elevated in intestinal biopsies from CD patients on GFD [43]. In dendritic cells from CD patients at all stages of the disease, more IL-15 has been found in the cell membranes, indicating that this key mediator of the immune response is constitutively altered in [77]. In CD patients at GFD, the IL-15R-α receptor has been observed at higher levels in intestinal biopsies [31].

In conclusion, the data in the literature point to several constitutive alterations of cell structure, signaling, proliferation and stress/innate immunity in CD cells (Figure 4). These pathways, already constitutively altered in celiac cells, render them more susceptible to the effects of the gliadin peptides that can act on the same pathways. These same three sets of metabolic pathways can be triggered in normal cells by P31–43, which mimics the celiac cellular phenotype in controls, as we will discuss in the next paragraph.

### 2.5. Celiac Cellular Phenotype Induced by Gliadin/P31–43 in Control Cells and Biopsies

**Figure 5:**
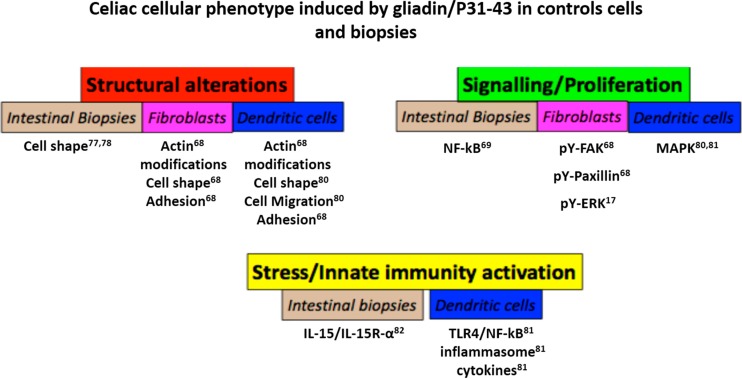
Celiac cellular phenotype induced by gliadin/P31–43 in control cells and biopsies. Schematic representation of the effects of gliadin peptide P31–43 on cells and biopsies from normal subjects. The main effects were grouped in three sets: structural alterations (actin modifications, cell shape, adhesion and cell migration), signaling/proliferation (NF-κB, pY-Fak, pY-paxillin, pY-ERK) and stress/innate immunity activation. In all cases, there was a quantitative increase in the markers cited, although in the case of actin, the alterations were qualitative. Numbers indicate the bibliographic references.

Several reports in the literature introduced the concept that gliadin may not be safe for non-celiac individuals (Figure 5).

#### 2.5.1. Structural Alterations

In the 1980s, it was reported that gluten could induce small intestinal mucosal structural alteration and symptoms in normal subjects, suggesting that gliadin may not be safe for non-celiac subjects [78,79].

More recently, we have demonstrated that the peptide P31–43 reproduces the structural alterations of the celiac cells in several cell types. These include actin modifications and cell shape alterations [17,80]. Remarkably, P31–43 could induce in control fibroblasts cell shape and actin modifications with alterations of focal adhesion and adhesion similar to the constitutive alterations described in celiac fibroblasts [68]. Moreover, in dendritic cells from control subjects, P31–43 can induce alterations of cell shape and motility [81] that mimic the alterations of cell shape and actin constitutively present in celiac dendritic cells [82].

#### 2.5.2. Cell Signaling and Proliferation

Exposure of the intestine of normal subjects to gliadin may cause the overall deregulation of NF-κB-related gene expression similar to the alteration of the pathway augmented in the CD intestine with and without gliadin treatment, although these alterations are more evident in the CD intestine [69].

Moreover, treatment with P31–43 can transiently increase EGFR staining of normal intestinal biopsies [83]. After treatment with P31–43, in fibroblasts and dendritic cells of normal subjects, many reports show an increase of several signaling pathways, including mitogen-activated protein kinase (MAPK) [8], ERK and other kinases similar to those constitutively altered in CD cells [17,68].

#### 2.5.3. Stress/Innate Immunity Activation

IL-15, the main mediator of the innate immunity response to gliadin in the celiac intestine, can be induced in intestinal biopsies of normal subjects by gliadin [84], confirming that gliadin is able to activate the same pathways found in celiac lesions in normal subjects. In control dendritic cells, gliadin can activate several cytokines and the inflammasome pathway [24,85]. Remarkably, P31–43 is able to increase IL-15 on the dendritic cell surface of normal subjects, mimicking the celiac dendritic cell phenotype [82].

Thus, gliadin is an activator of various signals at the cellular level mimicking the constitutive alterations found in celiac cells and intestinal biopsies. These pathways, constitutively altered in celiac cells, render them more susceptible to the effects of the gliadin peptides that, acting on the same pathways but in the celiac background, are able to produce long-term damage, including structural alterations, over-proliferation of crypt enterocytes and stress/innate immune response activation.

## 3. Conclusions

In the general issue of food and tissue inflammation, gliadin and its undigested peptides plays a leading role. In this paper, we have reviewed most of the effects of the gliadin peptide P31–43 in intestinal biopsies and cells from normal subjects and CD patients at different stages of the disease. We have highlighted the effects of an alimentary peptide, contained in very common foods, that is biologically active on cell structure, signaling/proliferation pathways and stress/innate immune activation. These are also constitutively altered in celiac cells and biopsies, rendering them more sensitive to the effects of gliadin.

What remains to be understood is the molecular defect explaining the alterations of the celiac cells, most likely due to a particular genetic make-up.

Gluten and other wheat proteins can induce inflammation in the intestine and outside. This also may occur in non-celiac subjects; *i.e.*, a particular fraction of wheat albumin, able to inhibit amylase and trypsin, can cause Toll-like receptor 4 (TLR4)-mediated intestinal inflammation [86].

Gluten itself could play a role in the pathogenesis of diseases different from CD, such as type 1 diabetes. In children with insulin-dependent diabetes but not celiac disease, intestinal inflammation [87,88] is triggered by viral infections and alimentary proteins [89]. In particular, signs of an altered mucosal immune response to gliadin have been described in type 1 diabetes both by challenging the rectum with gliadin peptides *in vivo* [90], and the proximal small intestine *in vitro* [91].

In conclusion, gliadin and its undigested peptides have biological effects not only in cells and the intestinal mucosa of patients with CD but also in normal subjects or in different diseases. How these effects can affect the health of non-celiac subjects will be the object of future research.
